# Improving Vaccine Knowledge Among Adolescents Aged 11–14 Years: A Pre–Post School-Based Educational Intervention

**DOI:** 10.3390/vaccines14050368

**Published:** 2026-04-22

**Authors:** Vincenza Sansone, Silvia Angelillo, Concetta Paola Pelullo, Francesca Gallè, Gabriella Di Giuseppe

**Affiliations:** 1 Department of Experimental Medicine, University of Campania “Luigi Vanvitelli”, 80138 Naples, Italygabriella.digiuseppe@unicampania.it (G.D.G.); 2 Department of Psychology and Health Sciences, Pegaso University, 80143 Naples, Italy; silvia.angelillo@unipegaso.it; 3 Department of Medical, Movement and Well-Being Sciences, University of Naples “Parthenope”, 80133 Naples, Italy; concettapaola.pelullo@uniparthenope.it

**Keywords:** adolescents, public health, school-based educational intervention, vaccination, vaccine literacy, vaccine knowledge

## Abstract

**Background/Objectives**: Schools may represent an ideal setting for increasing vaccine literacy and uptake. This quasi-experimental study took place between February and June 2025 with the aim of assessing the feasibility and effectiveness of a school-based educational intervention about vaccination among Italian adolescents. **Methods**: The European Commission’s e-Bug methodology was used to enhance vaccine knowledge in a sample of students attending four randomly chosen middle schools from Southern Italy. Pre and post-intervention vaccination knowledge was assessed through a questionnaire and compared through the Wilcoxon signed-rank test. Regression models were used to identify predictors of intervention-related outcomes. **Results**: A total of 262 students (mean age 12.3 ± 0.7 years, 52.3% female) participated in the study. A significant increase in vaccination knowledge score was registered from pre (5.6 ± 1.43) to post-intervention (6.79 ± 1.77). A significant improvement was found to be related to a lower number of cohabitants (OR = 0.61; 95% CI = 0.45–0.82), a lower score in the pre-test (OR = 0.60; 95% CI = 0.47–0.77), having considered the information provided completely clear (OR = 1.98; 95% CI = 1.05–3.74), and being willing to participate in similar future interventions (OR = 2.23; 95% CI = 1.12–4.42). **Conclusions**: These results show the effectiveness of school-based education strategies in increasing vaccine literacy within the targeted adolescent population. Similar interventions can be useful to increase compliance with vaccination in this age class. Randomized controlled studies are needed to confirm these findings.

## 1. Introduction

With more than 150 million deaths averted worldwide, it is estimated that immunization provided one of the greatest contributions to public health over the past 50 years [1,2]. Furthermore, more and more evidence is accumulating about the impact that vaccination against human papillomavirus has on preventing cervical cancer [3].

However, despite the success reached by vaccinology so far, vaccine uptake is still hindered by low confidence in vaccination, which can be related to demographic and socio-economic variables but also to misinformation and conspiracy theories [4]. Vaccine literacy (VL), which represents “people’s knowledge, motivation, and skills to find, understand, and evaluate immunization-related information in order to make adequate immunization decisions”, has been shown to be associated with educational level, socio-economic conditions, and migration background [5,6]. In Italy, where the anti-papillomavirus and the quadrivalent anti-meningococcal vaccinations are recommended, and the booster of diphtheria, tetanus, pertussis, and polio vaccines is mandatory for adolescents aged 11–18 [7,8], a survey performed in 2021 reported a rate of 32.4% of individuals with “limited” VL level [6]. During adolescence, individuals undergo significant life and social changes, seeking independence from parents and beginning to develop their own beliefs, attitudes, decisions, and habits [9]. Therefore, it is fundamental that interventions aimed at increasing vaccine confidence and uptake in this population group are designed considering these aspects. In particular, developing adolescents’ health literacy and decisional involvement regarding infections and vaccines is fundamental to increasing their confidence in vaccination and reducing their vaccine-related fear and anxiety [10]. A previous study performed among Italian adolescents found that a positive attitude towards vaccination was related to the usefulness of information received; notably, almost half of the participants believed that adolescents should have the autonomy to make their own vaccination decisions [11].

Schools may represent an ideal setting for increasing immunization rates. The literature shows that on-site vaccination events and education aimed at improving immunization knowledge can be successfully performed at school [10,12,13,14,15,16]. In particular, it has been shown that in-school education programs can improve students’ knowledge and attitude towards vaccines to a greater extent than community-based interventions [15]. As a matter of fact, vaccination is one of the topics of the health education programs included in the Health Promoting Schools national policies across several European countries [17].

Several approaches to improve vaccine knowledge and confidence have been used on different student populations and their parents so far, involving different professional figures, such as physicians or public health nurses, and sometimes vaccination clinics [10,12,15,16]. Some of these experiences were aimed at counteracting injection-related pain, fear, and stress-related actions, besides increasing knowledge [10,16].

The e-Bug program is an international partnership operated by the UK Health Security Agency, originally funded by the European Commission [18]. It promotes children’s and youth education about infection prevention and appropriate use of anti-microbials through the involvement of schools, parents and caregivers, and community leaders. The effectiveness and acceptance of its methods have been evaluated in several countries [19,20,21].

The goal of this study was to assess the feasibility and effectiveness of an educational intervention focused on vaccination and performed in the school setting among Italian adolescents. To this aim, a quasi-experimental approach based on the e-Bug program was used.

## 2. Materials and Methods

### 2.1. Setting and Participants

This study was conducted with a prospective quasi-experimental methodology and was part of a larger project investigating the role of education in preventing infectious diseases among adolescents [22]. The study was performed between February and June 2025 among a cohort of adolescents aged 11–14 attending middle schools in the Campania Region, Southern Italy. Performing a two-stage cluster sampling method, four institutions were randomly selected from the regional list of public schools, and in each school, the participating classes were randomly selected.

### 2.2. Collection Procedures

Before data collection, the principals were contacted to organize a first briefing with a member of the research group to describe the project, its objectives, and the methods to collect data. Once the principals’ authorization was obtained, a member of the research group handed each adolescent an envelope to take home with a written informed consent form and a cover letter describing the project. In particular, information was given on the aim of the study and on how to take part in it, the methodology to ensure data privacy and confidentiality, underscoring that participation was entirely voluntary. The students were requested to give the envelope to a parent or a legal guardian to sign the informed consent and authorize their participation. After ensuring that informed consent had been acquired, a member of the research group followed up after a ten-day interval to begin the project. Participants did not obtain any fees or gifts as compensation for their time spent participating in the project.

A pilot study including 50 students was performed to ensure the questionnaire’s clarity and feasibility. As no modifications were required, the pilot data were integrated into the overall analysis. A preliminary estimation of the standardized effect size (Cohen’s d = 0.61; 95% CI = 0.10), representing a medium-large effect, was assumed to calculate the a priori required sample size [23] from an initial subsample. The calculated sample size to realize the asked precision was 24 students, for an α = 0.05 (two-tailed) and 80% power.

The Ethics Committee of the University of Campania “Luigi Vanvitelli” approved the study (prot. N.0018199/i/01.07.2024). The project was conducted in accordance with the Declaration of Helsinki.

### 2.3. Educational Intervention

The project was developed and adapted to the Italian context from the e-Bug educational sections [22], focusing on Key Stage 3 students (11–14 years) on vaccination [18]. The contents focused on innate, acquired, and herd immunity and on the composition, methods of administration, and advantages of the vaccinations. The students were involved in interactive activities, gamification, and group discussions. The questionnaire contained four sections (Supplementary File S1). The first part explored socio-demographic and anamnestic characteristics, such as age, nationality, gender, non-communicable diseases, etc. The second section established baseline knowledge before the intervention (pre-test), while the third section evaluated knowledge immediately after the intervention (post-test) using the same items to evaluate the transformations in awareness.

Knowledge was measured with 10 multiple-choice items with four answer options on: the mechanism of immunity, how vaccinations are composed and administered, how a newborn receives antibodies, why vaccines are a preventive measure, and the advantages and disadvantages of vaccinations. The students were required to select only one answer that they considered most suitable. The fourth part of the questionnaire assessed the students’ perception of the educational intervention. The first three items evaluated the satisfaction, the perceived clarity, and the usefulness in daily choices of acquired knowledge using a 5-point Likert-type scale, where the score of “1” was assigned for “not at all” and “5” for “completely”. The fourth item investigated the need for additional information, and the last item explored the students’ willingness to participate in future projects, identifying the issues they would like to explore further.

The educational intervention was carried out in schoolrooms or in shared spaces (school gyms or auditoriums) by trained nurses who had completed a one-month training program focused on e-Bug methodology [18]. A 50 min lesson was conducted using slides made with child-friendly graphics and based on the e-Bug project materials (Supplementary File S2). In each intervention, at least three members of the research group were involved to train and check students, to lead a game using playing cards to stimulate herd immunity, and to conduct a group discussion focused on frequent vaccine misconceptions. In particular, each student was provided with a set of colored cards representing different health statuses: infected (red), recovering but infectious (blue), immune (white), and vaccinated (yellow). Students were initially assigned a “hidden” status of either “vaccinated” or “susceptible” (purple). The activity simulated the transmission of a pathogen over 7 days; when a “susceptible” student was touched by an “infected” peer, they progressed through the stages of infection (infected, recovering, and immune). Conversely, “vaccinated” students displayed their yellow card upon contact, effectively halting the transmission chain. By comparing the three scenarios, students could visually observe the downward trend in infection rates as vaccination coverage increased, providing a tangible demonstration of the herd immunity threshold [18].

Following the simulation, a structured group discussion titled “Reflecting Together” was conducted to consolidate the learning objectives. The discussion was centered on three key thematic questions: (1) why vaccination is not merely an individual choice but a collective public health responsibility; (2) the identification of vaccination as the primary strategic tool for the elimination of infectious diseases; (3) the multi-faceted importance of immunization for individual protection and community resilience. Facilitators guided the students to link their observations from the herd immunity game to these broader concepts, fostering a transition from experiential learning to conceptual understanding [18]. Finally, the fully filled-in questionnaires were collected. Take-home messages were shared with students with a final slide to recap the most important issues of the intervention. A card with a QR code linking to an online brochure to read and share with relatives was given to each student.

### 2.4. Statistical Analysis

To describe the socio-demographic data and the distribution of correct/incorrect answers for each knowledge item in both pre and post-tests, descriptive statistics (frequencies and percentages) were employed.

Two scores were derived by summing the number of correct responses at two specific intervals: pre-intervention test scores (“pre-test”) and post-intervention test scores (“post-test”). Scores were handled as continuous variables, and means and standard deviations were calculated for both pre and post-test scores.

The Shapiro–Wilk test revealed that the score differences did not follow a normal distribution, proving a *p* < 0.05. Thus, the non-parametric Wilcoxon signed-rank test was used to compare pre and post-test scores to determine whether the median of the differences was significantly different from zero. The threshold for statistical significance was set at α = 0.05, two-tailed. In addition, a paired t-test was done to complement the analysis; this was justified by the large sample size and the test’s robustness against normality violations [24,25]. The effect size r was calculated to quantify the intervention’s impact. For the analysis of individual dichotomous items (correct/incorrect), McNemar’s test was applied to track changes in knowledge.

Stepwise multivariate linear and logistic regression models were built to determine the predictors for four specific outcomes of interest: having higher post-intervention test scores (outcome 1, continuous); improved knowledge after the educational intervention (outcome 2, dichotomous); willingness to participate in similar future interventions (outcome 3, dichotomous); and considering the acquired knowledge completely useful for making daily health-related decisions (outcome 4, dichotomous). Adopting the Hosmer-Lemeshow model-building approach, variables were first assessed via bivariate analysis, and those reaching a value of *p* ≤ 0.25 were entered in the final models [26]. The following independent variable was tested in all models: having an employed mother (no = 0; yes = 1). The independent variable considering the information provided clear (not at all/slightly/moderately/very = 0; completely = 1) was tested in models 1, 2, and 4. The subsequent independent variables were tested in models 1 and 2: number of cohabitants (continuous), pre-intervention test scores (continuous), and willingness to participate in similar future interventions (no = 0; yes = 1). The following independent variables were tested in models 1 and 4: nationality (foreign = 0; Italian = 1) and being satisfied with the educational intervention (not at all/slightly/moderately/very = 0; completely = 1). The independent variable gender (female = 0; male = 1) was tested in models 1 and 3. The independent variable of considering the acquired knowledge useful for making daily health-related decisions (not at all/slightly/moderately/very = 0; completely = 1) was tested in model 1. Having a chronic medical condition (no = 0; yes = 1) was tested in model 2 as an independent variable. The following independent variables were tested in model 3: having parents with chronic medical conditions (no = 0; yes = 1), having a father employed (no = 0; yes = 1), and having improved knowledge after the educational intervention (no = 0; yes = 1). The independent variables age (continuous), having both parents with a university degree (no = 0; yes = 1), and post-intervention test scores (continuous) were tested in model 4. Results are reported as β coefficient or ORs and 95% confidence intervals (CIs). Statistical significance was defined as a two-tailed *p*-value ≤ 0.05. All statistical procedures were performed using STATA 17.

Model fitting for all outcomes was performed using a backward stepwise elimination procedure, with significance levels for entry and removal set at *p* < 0.20 and *p* > 0.40, respectively. For the continuous outcome (having higher post-intervention test scores), a multiple linear regression model was fitted. Model fitness was assessed through the coefficient of determination (R^2^) and the variance inflation factor (VIF) to rule out multicollinearity [27]. For the dichotomous outcomes (improved knowledge after the educational intervention, willingness to participate in similar future interventions, and considering the acquired knowledge completely useful for making daily health-related decisions), logistic regression models were employed. The goodness-of-fit for these models was evaluated using the Hosmer–Lemeshow test for calibration and the Area Under the ROC Curve (AUC) for discriminative power [28,29]. The Akaike Information Criterion (AIC) was reported for all models to ensure statistical parsimony.

## 3. Results

### 3.1. Characteristics of the Participants

A total of 262 adolescents provided consent to participate in the study out of an initial pool of 339, yielding a response rate of 77.3% (262/339). Table 1 details the main characteristics of the sample. The average age was 12.3 years; slightly more than half of the participants were female (136/260, 52.3%), and almost all were Italian (244/261, 93.5%); the mean of cohabitants was 3.2; and 21.6% (56/259) of adolescents reported at least one chronic medical condition. Regarding their parents, fewer than one-third had both parents with a university degree (29.1%, 71/244), two-thirds had both parents employed (69.3%; 169/244), and 25.1% (65/259) had a parent with a chronic medical condition.

### 3.2. Knowledge Related to Vaccination

Knowledge related to vaccination and the immune system was evaluated before the intervention. Overall, 75.2% (197/262) of adolescents correctly identified what the immune system is, and 62.7% (163/260) correctly understood the meaning of herd immunity. Exploring knowledge, merely 5% (13/261) of adolescents were aware of vaccine components, 54.4% (142/261) recognized that vaccination concerns both the individual and the entire community, and 86.6% (227/261) were aware of the risks of people not getting vaccinated (Table 2). After the intervention, 8 out of the 10 research questions showed an increase in the percentage of correct responses. In particular, 82.3% (209/251) of participants correctly identified what the immune system is, and 66.7% (168/252) correctly understood the meaning of herd immunity. Regarding knowledge, 38.8% (99/255) were aware of vaccine components, 69.6% (176/253) recognized that vaccination concerns both the individual and the entire community, and 91.7% (233/254) knew the risks of not getting vaccinated (Table 2).

The McNemar test indicated significant pre–post intervention changes for 4 of the 10 examined questions; complete results are reported in Table 2 and in the Supplementary File S3. In detail, responses to Q2 (How is a vaccine composed?), Q3 (How are vaccines administered?), Q4 (How does a newborn receive antibodies?), and Q9 (What is required to eliminate an infectious disease?) presented statistically significant increases.

Among the sample, the intervention led to significantly improved knowledge regarding vaccination, showing a mean of 5.6 ± 1.43 in the pre-intervention score, which increased to a mean of 6.79 ± 1.77 in the post-intervention score. A gender-based subgroup analysis revealed that both genders showed a significant increase in knowledge after the intervention. Male participants showed an increase in mean score from 5.44 ± 1.55 at the beginning to 6.59 ± 1.91 post-intervention, whereas females reported a mean pre-intervention score of 5.63 ± 1.34, which improved to 6.92 ± 1.67 in the post-intervention score. No statistically significant differences were observed among genders in either baseline (*p* = 0.3021) or post-intervention (*p* = 0.1528) knowledge.

Previously, by doing the final analysis, the normality of the difference between scores was measured by the Shapiro–Wilk test, which showed a significant deviation from normality (*p* = 0.003). Therefore, the non-parametric Wilcoxon signed-rank test evaluated pre- and post-intervention scores. Results revealed a statistically significant improvement in post-intervention scores (Z = 9.11, *p* < 0.001). The assessed effect size (r = 0.673) indicated a medium-to-large magnitude of improvement, showing a substantial increase after the intervention in adolescents’ knowledge, endorsed by the paired t-test (t = −10.542; *p* < 0.001). Reliable results from both the Wilcoxon signed-rank test and the paired t-test proved the strength of the results.

The results of the multivariate linear and logistic regression analysis conducted to explore the factors that could be associated with the different outcomes of interest are shown in Table 3. The multivariate linear regression model showed that adolescents who reported having higher pre-intervention test scores, those who had a smaller number of cohabitants, those who considered the information provided completely clear, and those who were willing to participate in similar future interventions were more likely to achieve higher post-intervention test scores (model 1 in Table 3 and Supplementary File S4). The linear regression for model 1 showed an R^2^ of 0.25, with no evidence of multicollinearity (mean VIF = 1.02).

Data from a multivariate logistic regression model demonstrated a significant improvement of knowledge after the educational intervention among participants with a lower number of cohabitants (OR = 0.61; 95% CI = 0.45–0.82), those who had a lower score in the pre-test (OR = 0.60; 95% CI = 0.47–0.77), those who considered the information provided completely clear (OR = 1.98; 95% CI = 1.05–3.74), and those willing to participate in similar future interventions (OR = 2.23; 95% CI = 1.12–4.42) (model 2 in Table 3 and Supplementary File S5). Model 2 demonstrated good calibration (Hosmer–Lemeshow *p* = 0.2953) and discriminative power (AUC = 0.7568).

### 3.3. Adolescents’ Perception of the Intervention

More than half of the sample population declared to be fully satisfied with the educational intervention (56.8%; 142/240), with no significant difference between genders (58.5—69/118—for males and 56.2%—73/130—for females) (*p* = 0.712). Additionally, 63.4% (158/249) considered the information provided to be completely clear, while 55.6% (138/248) considered the acquired knowledge completely useful for making daily health-related decisions. Additionally, more than one-fourth (27.5%; 66/240) of adolescents stated a need for additional information on vaccination, and 75.4% (184/244) indicated a willingness to participate in similar future interventions. The multivariate logistic regression used to analyze the key factors influencing participants’ willingness to participate in similar future educational interventions indicated that females (OR = 1.97; 95% CI = 1.01–3.84) and those whose mothers were employed (OR = 2.16; 95% CI = 1.09–4.27) were more willing to participate (model 3 in Table 3 and Supplementary File S5). Model 3 was well-calibrated (Hosmer–Lemeshow *p* = 0.3165; AUC = 0.6534). Finally, the multivariate logistic regression model showed that the youngsters (OR = 0.58; 95% CI = 0.36–0.95), those who considered the information provided completely clear (OR = 5.71; 95% CI = 2.77–11.77), and those fully satisfied with the educational intervention (OR = 4.13; 95% CI = 2.08–8.19) were more likely to consider the acquired knowledge completely useful for making daily health-related decisions (model 4 in Table 3 and Supplementary File S5). Finally, model 4 exhibited excellent model fitness, with a Hosmer–Lemeshow *p*-value of 0.4831 and an AUC of 0.8381.

## 4. Discussion

Our study evaluates the feasibility and effectiveness of a school-based educational intervention on vaccination among Italian adolescents aged 11–14 years, using a quasi-experimental approach with an adapted form of the e-Bug program. Overall, our findings underscore the importance of schools as settings for promoting VL during adolescence, indicating that the intervention was well accepted and effective in improving students’ knowledge of vaccination. The present study contributes to the existing literature, providing evidence from Southern Italy, a context in which data on adolescent VL are still limited and where a proportion of the general population has been reported to have limited VL levels [6].

After the intervention, a significant improvement in overall knowledge scores was observed in both males and females, with no gender-based differences in baseline or post-intervention knowledge. This finding is consistent with previous school-based educational studies conducted among adolescent populations, which have reported improvements in vaccine-related knowledge and VL following structured educational interventions in the school setting [10,15]. In particular, the McNemar test showed statistically significant improvements in specific domains such as vaccine composition, administration, and the role of vaccination programs in disease elimination.

Moreover, baseline knowledge was already relatively high for some aspects, which could reflect the inclusion of vaccination topics in public health campaigns in Italy, particularly after the COVID-19 pandemic. Nevertheless, the observed post-intervention improvements suggest that interactive and age-specific educational approaches can consolidate the level of knowledge, particularly regarding more complex concepts that are frequently misunderstood among adolescents [11]. These domains have previously been reported to be poorly understood among adolescents, as also reported in studies conducted in Italy and other European countries [11,20]. Indeed, the increase in correct responses for these items observed in our study suggests that more interactive and age-tailored educational approaches, such as those used in the e-Bug program, may be effective in addressing these topics, which are rarely covered in depth in standard school curricula. The presented results are in line with previous studies indicating that structured, school-based educational interventions could significantly improve adolescents’ knowledge and literacy related to vaccination [10,15].

Multivariate analyses highlighted several factors associated with higher post-intervention test scores. Students with higher baseline knowledge achieved higher post-test scores, whereas those with lower pre-test scores were more likely to show improvement; these aspects suggest that the intervention was effective across different initial knowledge levels. This finding is consistent with previous studies indicating that educational interventions could reinforce existing knowledge and improve understanding among students with lower baseline knowledge [15,30]. Interestingly, a lower number of cohabitants was associated with higher post-intervention test scores and a greater knowledge improvement; it may reflect differences in parental attention or access to health-related information and is consistent with evidence indicating that socio-demographic and family-related factors are important determinants of VL, potentially influencing adolescents’ ability to access and retain health information and, consequently, the effectiveness of educational interventions [6].

One of the most relevant findings of our study is the association between perceived clarity of the information acquired during the intervention and the outcomes, including higher post-intervention test scores, improved knowledge, and perceived usefulness of the acquired knowledge for daily health-related decisions. This result aligns with previous studies indicating that clarity and comprehensibility of vaccine information are key determinants of VL and confidence [5,6]. This highlights the importance of providing clear, age-appropriate information when addressing health topics, such as vaccination, among adolescents.

The association between higher post-intervention knowledge and willingness to participate in future interventions suggests that motivation is a key factor in educational impact. A similar result was reported in studies using participatory approaches and gamification, where students improved their knowledge and acceptance of vaccination programs [13,16,19].

Moreover, the high levels of satisfaction (56.8%) reported by participants and their interest in future school-based health promotion interventions (75.4%) support the acceptability of the initiative. Similar levels of student engagement and satisfaction have been reported in other school-based vaccination education programs, in particular those that include an interactive approach, gamification, and peer discussion [13,16]. These approaches seem particularly effective during adolescence, a delicate phase of life characterized by sensitivity to peer influence and increasing autonomy and critical thinking.

The intervention was considered positively by participants, with more than half reporting satisfaction (56.8%) and considering the information completely clear (63.4%) and useful for daily health-related decisions (55.6%). These results are in line with other studies using the e-Bug program, which reported a high level of acceptability among students across different contexts and countries [20,21]. Furthermore, the multivariate analysis showed that younger students and those who were completely satisfied with the educational intervention were more likely to consider the acquired knowledge completely useful for making daily health-related decisions. The association between younger age and positive perception may suggest that younger adolescents are more receptive to health education messages and more inclined to integrate newly acquired information into their daily choices. Being completely satisfied with the educational intervention may indicate the role of engagement and positive learning experience in enhancing the perceived value of health education, as higher satisfaction may reflect greater involvement during the intervention, as also suggested in previous school-based studies [13,16].

Moreover, the high percentage of students willing to participate in similar future interventions (75.4%) supports the need to integrate vaccination education into school activities. This finding is particularly relevant in light of the Health Promoting Schools agenda [31], and, in particular, with the project School for Health in Europe [17], which emphasizes the role of schools in promoting health literacy and preventive behaviors among children and adolescents. Female students were more likely to express willingness to participate in future educational interventions, and maternal employment was also positively associated with this outcome. Gender differences in health-related attitudes have been reported in previous studies on health education and preventive behaviors among adolescents [10,11]. Moreover, the association with maternal employment may indicate that socio-economic or cultural factors influence health awareness within families through educational initiatives, although this warrants further investigation.

The results of this study reinforce the idea that VL is a multidimensional construct that goes beyond the acquisition of knowledge. This perspective is consistent with the analysis proposed by Badua et al., which frames VL as a part of health literacy and involving the ability to understand, evaluate, and apply vaccination-related information [5]. Indeed, by actively involving adolescents in health issues, such as vaccines, and addressing common misconceptions, school-based programs may not only increase knowledge but also promote responsible health behaviors. This is particularly relevant in the context of vaccine hesitancy and misinformation, which continue to reduce vaccine uptake globally [4,32]. From a public health perspective, improving VL may represent an important strategy to strengthen preventive medicine initiatives and to increase awareness about vaccination.

Furthermore, this study underscores the role of health education in promoting positive attitudes towards vaccination, essential for improving vaccine uptake. Given that a proportion of students need additional information, integrating such programs within the school curricula could represent an important approach to neutralize misinformation and vaccine hesitancy, which remain significant barriers to achieving high immunization coverage [4].

Since the e-Bug materials used are standardized and available in multiple languages, the intervention is highly reproducible in different geographic areas, ensuring external validity of the findings for middle school settings in similar socio-economic and cultural contexts. However, while the core predictors of knowledge improvement (such as the clarity of the intervention and initial knowledge levels) are likely universal, the specific influence of parental occupation may vary in regions with different socio-economic structures. Overall, the study provides a scalable model for school-based vaccination education that can be adapted to various national health curricula.

This study has several limitations that merit discussion. First, the quasi-experimental design without a control group limits the ability to attribute improvements in knowledge solely to the educational intervention, given that external factors may have influenced the outcomes. To mitigate this, we utilized a standardized, internationally validated curriculum (e-Bug), which has previously shown consistent efficacy in similar populations, thus reducing the likelihood that observed gains were entirely due to external factors. Second, although self-reported questionnaires can be subject to social desirability bias, they ensured total anonymity and focused on objective knowledge-based questions rather than purely attitudinal scales, which are more susceptible to such bias. Third, the follow-up was limited to the immediate post-intervention period; it was not possible to make a long-term evaluation of knowledge or VL. While this allows for an assessment of immediate knowledge acquisition, it does not account for long-term retention. To address this, we focused the educational session on core conceptual understanding (e.g., the mechanics of herd immunity) rather than rote memorization, as conceptual knowledge is typically more stable over time. Further investigations could assess the durability of these outcomes and the long-term impact on VL.

Despite its limitations, this study presents several strengths. First, the educational intervention was based on the e-Bug curriculum, an internationally recognized and scientifically validated framework for school-based health education. Second, the use of active learning strategies (gamification and group discussions) ensured high student engagement, moving beyond traditional front-of-class lecturing. Third, targeting the 11–14 age group is strategic and critical, as adolescents in this developmental stage are beginning to transition from passive recipients of healthcare decisions to autonomous actors. By providing evidence-based information at this time, students develop their critical thinking skills, necessary to navigate future health choices and counter misinformation about vaccinations.

## 5. Conclusions

In conclusion, our findings support the idea that the integration of interactive and clear vaccination education into middle school is a strategy to enhance VL during a critical developmental period. Therefore, educational interventions should not be grounded solely in knowledge; they should also promote critical thinking to build greater trust in health information. Moreover, integrating parents into school-based programs may enhance effectiveness, given that family context is important in adolescent vaccination decisions. Further research is needed to better understand how educational interventions can be integrated into school curricula to improve VL among adolescents, with a randomized controlled trial design to assess the effectiveness of such educational interventions and also to evaluate their long-term impact on vaccination behaviors across the life course.

## Figures and Tables

**Table 1:** Socio-demographic and anamnestic characteristics of the study population.

Characteristics		
*Socio-demographic*		
	Mean ± Standard Deviation (Range)	
**Age, years (continuous)**	12.3 ± 0.7 (11–14)	
**Number of cohabitants (258) ^a^**	3.2 ± 1.1 (1–9)	
	**N**	**%**
**Gender (260) ^a^**		
Male	124	47.7 (124/260)
Female	136	52.3 (136/260)
**Nationality (261) ^a^**		
Italian	244	93.5 (244/261)
Foreign	17	6.5 (17/261)
**Mother with a university degree (253) ^a^**		
No	143	56.5 (143/253)
Yes	110	43.5 (110/253)
**Father with a university degree (247) ^a^**		
No	156	63.2 (156/247)
Yes	91	36.8 (91/247)
**Mother employed (253) ^a^**		
No	77	30.4 (77/253)
Yes	176	69.6 (176/253)
**Father employed (248) ^a^**		
No	5	2 (5/248)
Yes	243	98 (243/248)
***Anamnestic***		
**Chronic medical condition (259) ^a^**		
No	203	78.4 (203/259)
Yes	56	21.6 (56/259)
**Parents’ chronic medical condition (259) ^a^**		
No	194	74.9 (194/259)
Yes	65	25.1 (65/259)

^a^ Number of respondents for each item.

**Table 2:** Pre and post-intervention knowledge levels with corresponding McNemar test results.

Knowledge	Pre-Intervention Correct	Post Intervention Correct	McNemar		
	N (%)	N (%)	χ^2^	95% CI *	*p* Value
Q1. What is the immune system?	197 (75.2)	209 (82.3)	4.19	0.36–1.01	0.053
Q2. How is a vaccine composed?	13 (5)	99 (38.8)	73.96	0.03–0.16	<0.001
Q3. How are vaccines administered?	197 (75.2)	253 (66.4)	96.99	0.04–0.15	<0.001
Q4. How does a newborn receive antibodies?	29 (11)	202 (79.5)	36.51	0.12–0.38	<0.001
Q5. Why is a vaccine considered a preventive measure?	201 (77)	193 (76)	0.16	0.64–1.94	0.791
Q6. Do vaccinations concern both the individual and the entire community?	142 (54.4)	176 (69.6)	1.64	0.69–4.81	0.286
Q7. What happens when people do not get vaccinated?	227 (86.6)	233 (91.7)	2.63	0.28–1.17	0.143
Q8. What happens when the number of vaccinated people increases?	224 (85.8)	210 (83)	1.19	0.76–2.42	0.341
Q9. What is required to eliminate an infectious disease?	201 (77)	217 (85.4)	6.48	0.24–0.88	0.015
Q10. What is herd immunity?	163 (62.7)	168 (66.7)	1.43	0.45–1.23	0.282

* CI = Confidence Interval.

**Table 3:** Results of the linear and logistic regression analysis showing the factors associated with the outcomes of interest.

***Model 1. Having higher post-intervention test scores*** *F (5, 217) = 14.1, R^2^ = 25%, adjusted R^2^ = 23%, p < 0.0001, AIC 832.09*				
**Variables**	**Coeff**	**SE**	**t**	***p***
Pre-intervention test scores	0.46	0.07	6.29	<0.001
Considered the information provided to be completely clear	0.73	0.22	3.40	0.001
Having a lower number of cohabitants	−0.27	0.09	−2.95	0.004
Willingness to participate in similar future interventions	0.53	0.24	2.18	0.030
Italian nationality	−0.66	0.42	−1.57	0.117
***Model 2. Improved knowledge after the educational intervention*** *Log likelihood = −121.84356, χ2 =42.45 (df = 5), p < 0.0001, AIC 255.69*				
	**OR**	**95%CI**		***p***
Pre-intervention test scores	0.60	0.47–0.77		<0.001
Having a lower number of cohabitants	0.61	0.45–0.82		0.001
Willingness to participate in similar future interventions	2.23	1.12–4.42		0.022
Considered the information provided to be completely clear	1.98	1.05–3.74		0.034
Having at least one chronic medical condition	1.47	0.69–3.15		0.322
***Model 3. Willingness to participate in similar future interventions*** *Log likelihood = −108.61158, χ2 = 15.11 (df = 4), p < 0.0001, AIC 227.22*				
Having a mother employed	2.16	1.09–4.27		0.027
Women	1.97	1.01–3.84		0.046
Improved after intervention	1.88	0.96–3.7		0.066
Having a father employed	3.46	0.43–27.59		0.241
***Model 4. Considering the acquired knowledge is completely useful for making daily health-related decisions*** *Log likelihood = −109.27168, χ2 = 88.62 (df = 5), p < 0.0001, AIC 230.54*				
Considered the information provided to be completely clear	5.71	2.77–11.77		<0.001
Completely satisfied with the educational intervention	4.13	2.08–8.19		<0.001
Younger	0.58	0.36–0.95		0.030
Having parents with a university degree	1.72	0.82–3.6		0.149
Mother employed	1.65	0.79–3.42		0.178

## Data Availability

The data presented in this study are available upon request from the corresponding author.

## Supplementary Materials

The following supporting information can be downloaded at: https://www.mdpi.com/article/10.3390/vaccines14050368/s1, Supplementary File S1: Questionnaire; Supplementary File S2: Cards game; Supplementary File S3: Grouped bar chart illustrating the percentage of correct responses for each of the ten knowledge items (Q1–Q10) before and after the intervention; Supplementary File S4: The coefficient plot for linear regression displays the standardized coefficients and 95% confidence intervals for predictors of the post-intervention test score; Supplementary File S5: The coefficient plot illustrates the Odds Ratios (OR) and 95% confidence intervals from the logistic regression model.
