# Supplementary material for: Improving Vaccine Knowledge Among Adolescents Aged 11–14 Years: A Pre–Post School-Based Educational Intervention
Source: Vaccines (Basel). 2026 Apr 22;14(5):368. doi: 10.3390/vaccines14050368 (PMC13211575; doi:10.3390/vaccines14050368)
Supplement: Supplementary file 1 [file vaccines-14-00368-s001.zip › File S1.pdf]

Supplementary File S1. Questionnaire.

SECTION A: SOCIO-DEMOGRAPHIC AND ANAMNESTIC CHARACTERISTICS

A1. What is your gender? □ Male □ Female

A2. How old were you on your last birthday? __________

A3. What is your nationality? □ Italian □ Other, please specify: __________

A4. Which school do you attend? __________

A5. Which grade are you in? □ First □ Second □ Third

A6. How many people live in your household? __________

A7. What is your mother's highest educational level?

□ None □ Elementary school □ Middle school □ High school □ University degree

A8. What is your father's highest level of education?

□ None □ Elementary school □ Middle school □ High school □ University degree

A9. What is your mother's occupation? □ None □ Other, please specify: __________

A10. What is your father's occupation? □ None □ Other, please specify: __________

A11. Do you have any chronic medical conditions?

□ No □ Yes, (please specify, more than one disease is allowed) __________

A12. Do your parents have any chronic medical conditions?

□ No □ Yes, (please specify, more than one disease is allowed) __________

SECTION B. PRE-TEST: Please read the questions and mark the answer you consider correct

	**A**	**B**	**C**	**D**
**B1.** What is the immune system?	□ Being immune means being protected against the severe effects of infections	□ Being immune means never getting a disease	□ Being immune means not being protected from severe disease	□ None of the above
**B2.** How is a vaccine composed?	□ A small amount of inactive and harmless microorganisms	□ A large amount of inactive and harmless microorganisms	□ A small amount of active and harmless microorganisms	□ A large amount of active and harmless microorganisms
**B3.** How are vaccines administered?	□ By injection	□ Orally	□ As eye drops	□ A + B
**B4.** How does a newborn receive antibodies?	□ During pregnancy	□ Through breastfeeding	□ A + B	□ None of the above
**B5.** Why is a vaccine considered a preventive measure?	□ It strengthens the body's immune defenses	□ It treats the disease	□ It triggers the onset of the disease	□ None of the above
**B6.** Vaccinations concern:	□ The individual	□ The entire community	□ A + B	□ None of the above
**B7.** What happens when people do not get vaccinated?	□ Infectious diseases become more widespread	□ Nothing changes	□ Infectious diseases become less widespread	□ The body becomes stronger
**B8.** What happens when the number of vaccinated people increases?	□ Infections spread easily	□ It becomes extremely difficult for infections to spread	□ There is no difference in the spread of infections	□ All of the above
**B9.** What is required to eliminate an infectious disease?	□ A vaccination program targeting only vulnerable individuals	□ A vaccination program accessible to the entire population	□ A vaccination program targeting only healthcare workers	□ A vaccination program targeting only adolescents
**B10.** What is herd immunity?	□ Immunity acquired after contracting an infection	□ Immunity acquired through vaccination	□ Immunity that occurs when few individuals are vaccinated or infected	□ Immunity that occurs when an unvaccinated individual is protected because most people around them are vaccinated

SECTION C. POST-TEST: Please read the questions and mark the answer you consider correct				
	**A**	**B**	**C**	**D**
**C1.** What is the immune system?	□ Being immune means being protected against the severe effects of infections	□ Being immune means never getting a disease	□ Being immune means not being protected from severe disease	□ None of the above
**C2.** How is a vaccine composed?	□ A small amount of inactive and harmless microorganisms	□ A large amount of inactive and harmless microorganisms	□ A small amount of active and harmless microorganisms	□ A large amount of active and harmless microorganisms
**C3.** How are vaccines administered?	□ By injection	□ Orally	□ As eye drops	□ A + B
**C4.** How does a newborn receive antibodies?	□ During pregnancy	□ Through breastfeeding	□ A + B	□ None of the above
**C5.** Why is a vaccine considered a preventive measure?	□ It strengthens the body's immune defenses	□ It treats the disease	□ It triggers the onset of the disease	□ None of the above
**C6.** Vaccinations concern:	□ The individual	□ The entire community	□ A + B	□ None of the above
**C7.** What happens when people do not get vaccinated?	□ Infectious diseases become more widespread	□ Nothing changes	□ Infectious diseases become less widespread	□ The body becomes stronger
**C8.** What happens when the number of vaccinated people increases?	□ Infections spread easily	□ It becomes extremely difficult for infections to spread	□ There is no difference in the spread of infections	□ All of the above
**C9.** What is required to eliminate an infectious disease?	□ A vaccination program targeting only vulnerable individuals	□ A vaccination program accessible to the entire population	□ A vaccination program targeting only healthcare workers	□ A vaccination program targeting only adolescents
**C10.** What is herd immunity?	□ Immunity acquired after contracting an infection	□ Immunity acquired through vaccination	□ Immunity that occurs when few individuals are vaccinated or infected	□ Immunity that occurs when an unvaccinated individual is protected because most people around them are vaccinated

**SECTION D. EDUCATIONAL NEEDS REGARDING VACCINES**					
**Please respond by assigning a score from 1 (not at all) to 5 (completely).**					
**D1. How satisfied are you with the overall educational intervention?**	**1**	**2**	**3**	**4**	**5**
**D2. Do you consider the information provided to be clear?**	**1**	**2**	**3**	**4**	**5**
**D3. Do you consider the acquired knowledge useful for making daily health-related decisions?**	**1**	**2**	**3**	**4**	**5**
**D4. Do you need further information about vaccines?** □ No □ Yes					
**D5. If your school offered similar interventions in the future, would you be interested in participating?** □ No □ Not sure □ Yes<br>If yes, which topic would interest you? (please specify): ________________________					
