# Supplementary material for: Improving Vaccine Knowledge Among Adolescents Aged 11–14 Years: A Pre–Post School-Based Educational Intervention
Source: Vaccines (Basel). 2026 Apr 22;14(5):368. doi: 10.3390/vaccines14050368 (PMC13211575; doi:10.3390/vaccines14050368)

Supplementary File S2. Cards game

gb_ks3_v_sh1-5_herd_immunity_scenario_coloured_cards.docx

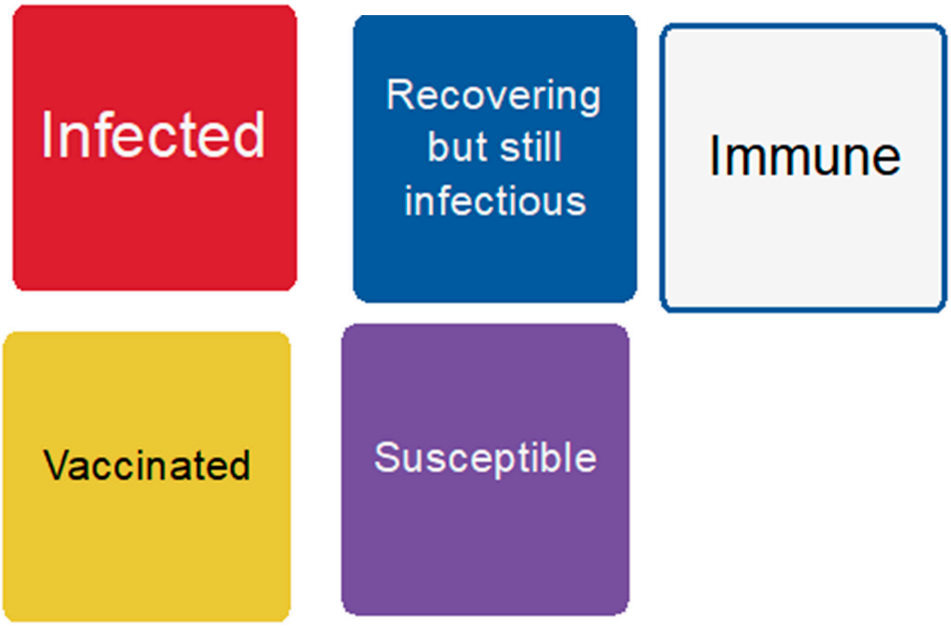

Slides

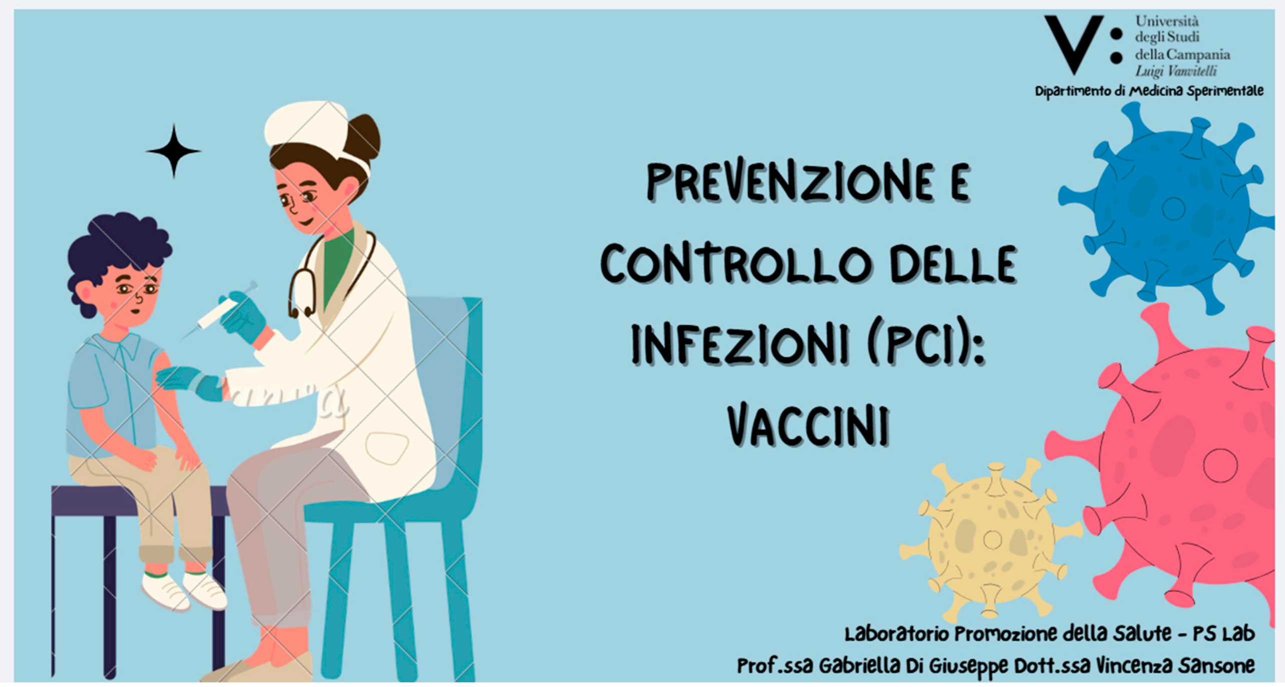
Università
degli Studi
della Campania
Luigi Vanvitelli
Dipartimento di Medicina Sperimentale
PREVENZIONE E CONTROLLO DELLE INFEZIONI (PCI): VACCINI
Laboratorio Promozione della Salute - PS Lab
Prof.ssa Gabriella Di Giuseppe Dott.ssa Vincenza Sansone

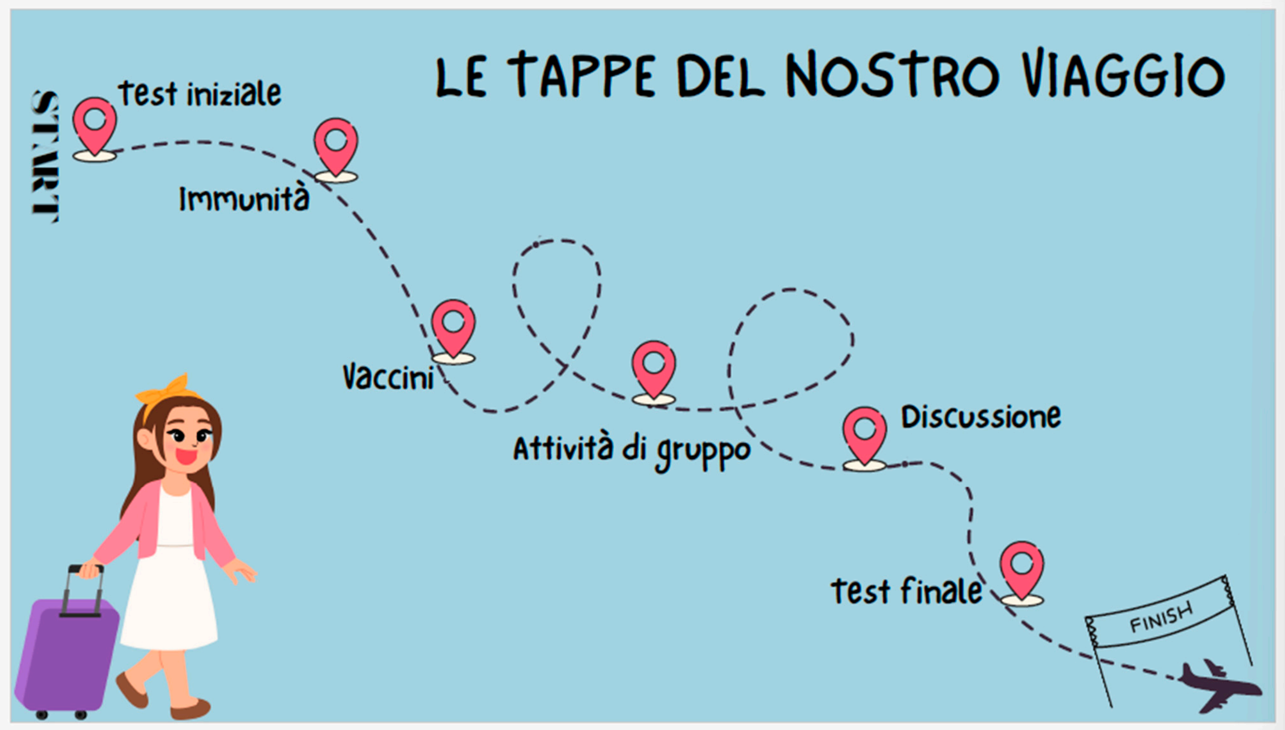
LE TAPPE DEL NOSTRO VIAGGIO
START
test iniziale
Immunità
Vaccini
Attività di gruppo
Discussione
test finale
FINISH

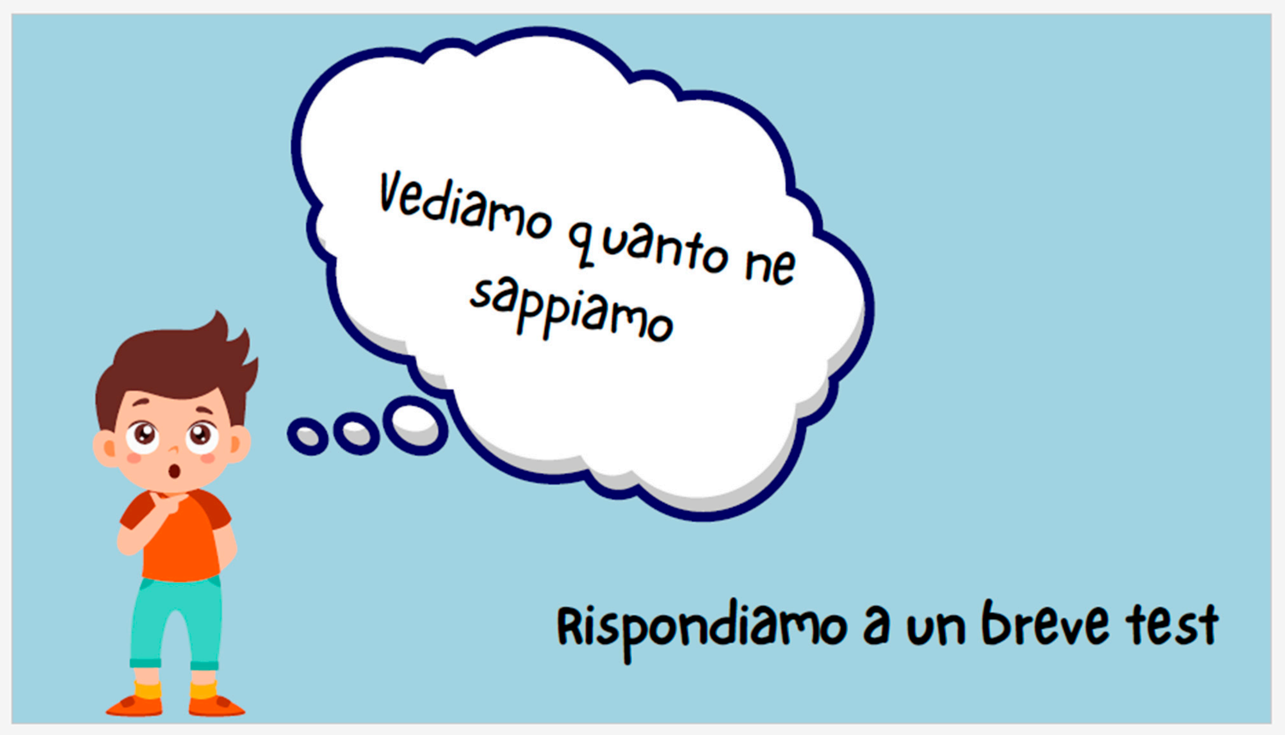
Vediamo quanto ne sappiamo
Rispondiamo a un breve test

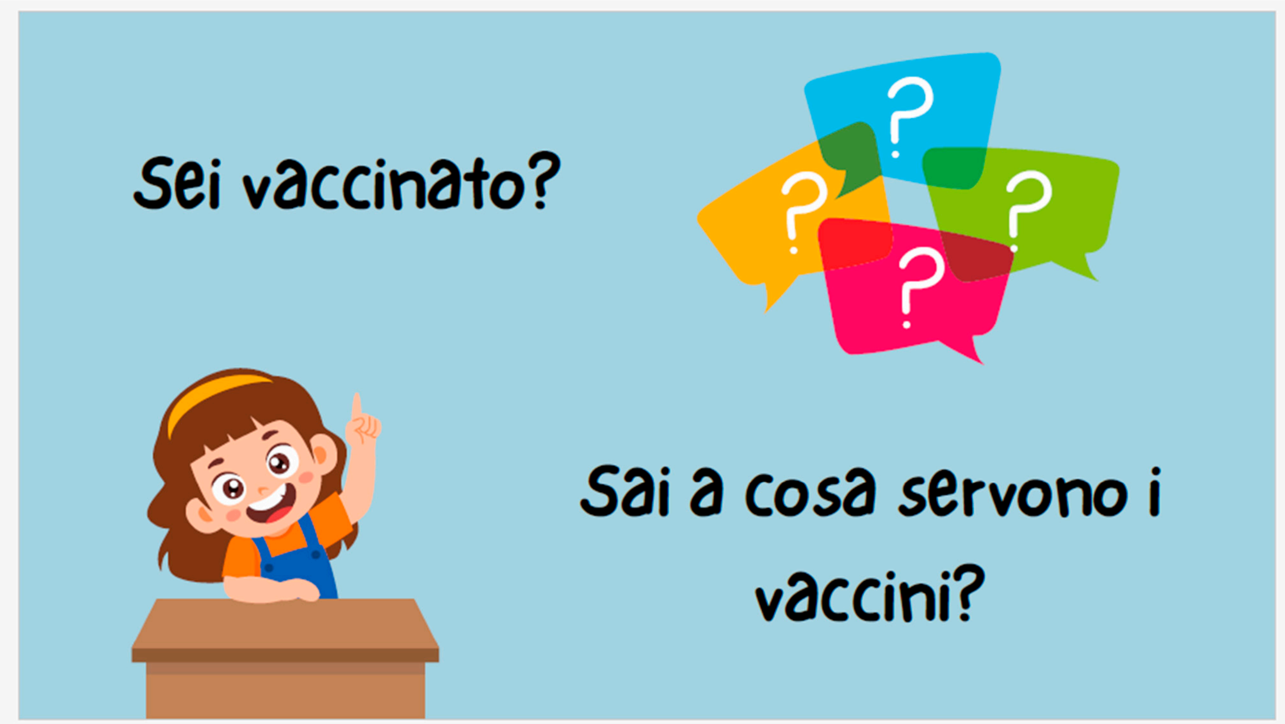
Sei vaccinato?
Sai a cosa servono i vaccini?

CHE COS'È IL SISTEMA IMMUNITARIO?

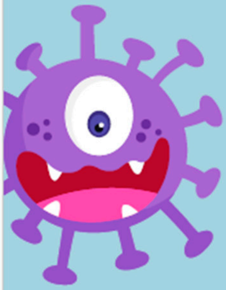

Il sistema immunitario è costituito da cellule speciali, proteine, tessuti e organi e ha il compito di protegge il nostro corpo dall'assalto di "invasori esterni" come batteri, virus e funghi.

AIFA 2023

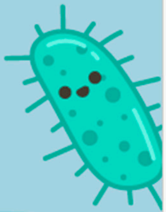

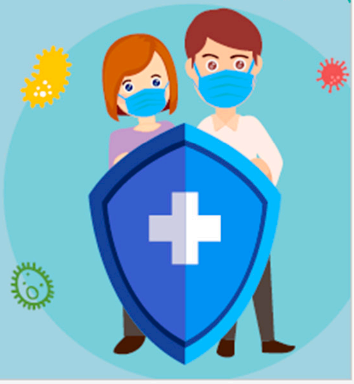

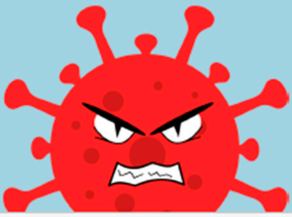

CHE COSA SIGNIFICA ESSERE IMMUNE?

Essere immune significa essere protetti dagli effetti gravi delle infezioni

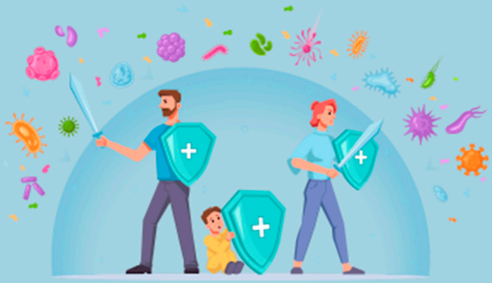

COME POSSO ESSERE IMMUNE?

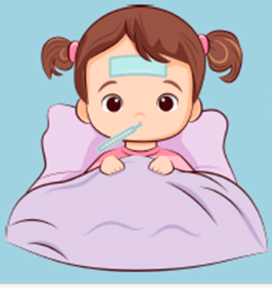

Contraendo la malattia e guarendo

Vaccinandomi

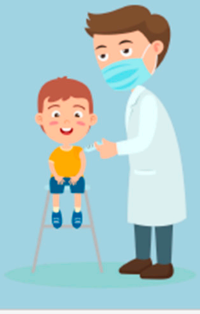

CHE COS'È UN VACCINO?

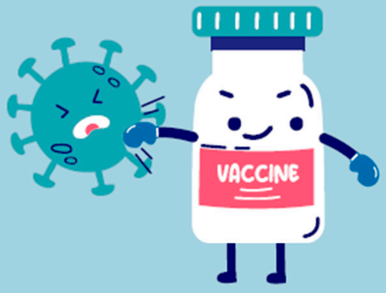

I vaccini sono medicinali biologici che hanno lo scopo di prevenire una o più malattie infettive attraverso la stimolazione del sistema immunitario e la conseguente acquisizione della cosiddetta "immunità attiva".

AIFA 2024

A COSA SERVONO I VACCINI?

Prevengono le Malattie: Proteggono da malattie gravi come il morbillo, la meningite, l'HPV ecc...

Proteggono il Gruppo: Aiutano a proteggere la comunità, creando l'immunità di gregge

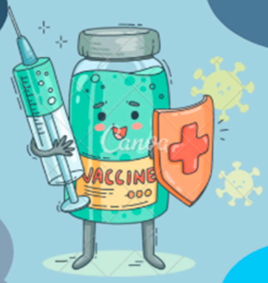

Riducono i Sintomi: Anche se si contrae la malattia, i sintomi sono generalmente meno gravi

Sicurezza: Contribuiscono a una vita più sana e sicura

COME SI SOMMINISTRA UN VACCINO?

tramite una puntura

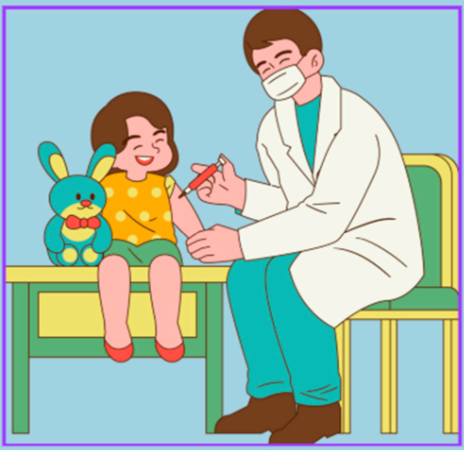

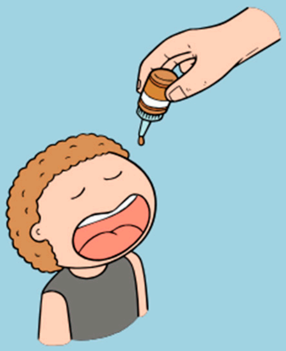

tramite farmaci da bere

MA UN VACCINO IN FONDO DA COSA È COMPOSTO?

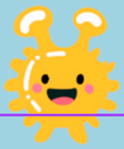

I vaccini sono composti da una piccola quantità di microrganismi inattivi e innocui, che insegnano al nostro corpo come combattere i microbi nocivi quando e se veniamo attaccati dalla malattia

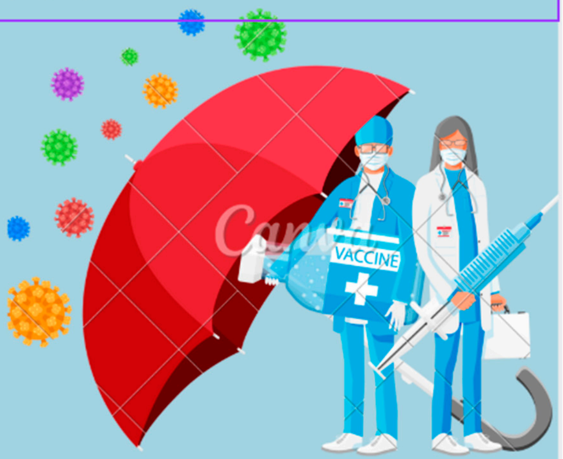

COME FUNZIONA UN VACCINO?

I vaccini stimolano il sistema immunitario a produrre anticorpi che combattono specifici microbi, senza farci ammalare

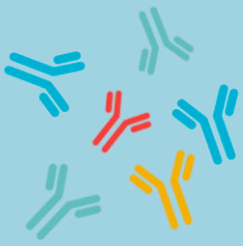

Durante la gravidanza, invece, la mamma dona i suoi anticorpi al bambino

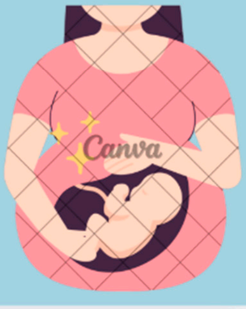

Dopo la nascita, gli anticorpi vengono trasmessi attraverso il latte materno, in questo modo il neonato è protetto dalle infezioni

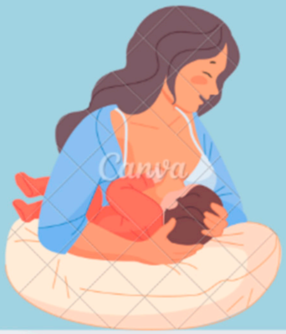

CALENDARIO VACCINALE IN ITALIA
ETÀ 11-14 ANNI

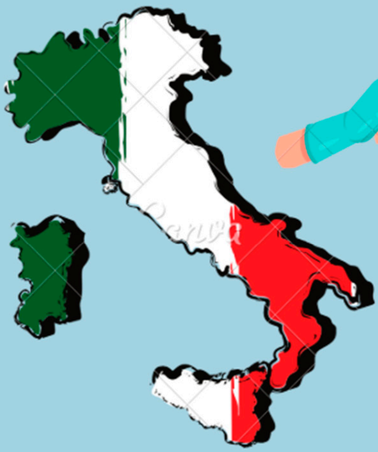

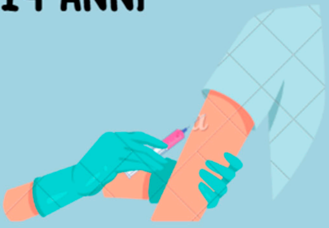

Vaccinazioni anti-difterite, tetano, pertosse, poliomielite
- Richiami dal compimento dei 12 anni di vita

Vaccinazione anti-papillomavirus
- 2 dosi dal compimento degli 11 anni di vita

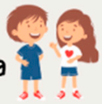

Vaccinazione anti-meningococco quadrivalente ACW135Y
- 1 dose tra il 12° e il 18° anno di vita

Vaccinazione anti-meningococco B
in base alla situazione epidemiologica della Regione Campania
- 2 dosi a intervallo di un mese

Piano Nazionale Prevenzione Vaccinale 2023-2025

IMPARIAMO GIOCANDO

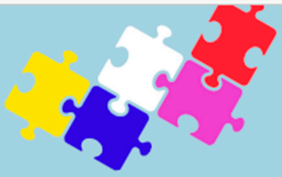

Legenda:

CARTA ROSSA soggetto infetto

CARTA BIANCA soggetto immune

CARTA BLU soggetto in via di guarigione ma ancora contagioso

CARTA GIALLA soggetto vaccinato

CARTA VIOLA soggetto sensibile

COS'È L'IMMUNITÀ DI GREGGE?

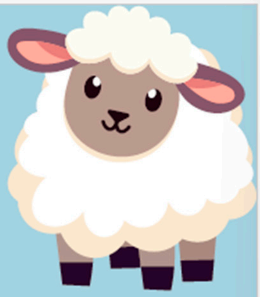

L'immunità di gregge è una protezione indiretta da malattie contagiose che si verifica quando una parte significativa della popolazione diventa immune, attraverso la vaccinazione o l'infezione naturale.

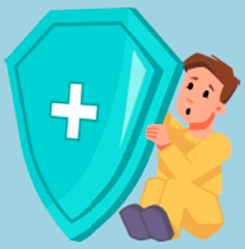

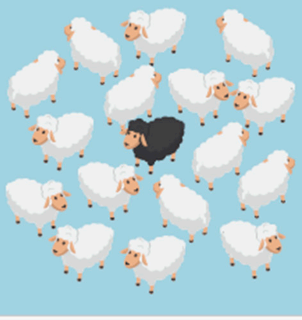

Questa immunità diffusa crea una barriera che ostacola la trasmissione della malattia, proteggendo anche gli individui non immuni o coloro che non possono vaccinarsi

TUTTI POSSONO AGIRE PER L'IMMUNIZZAZIONE

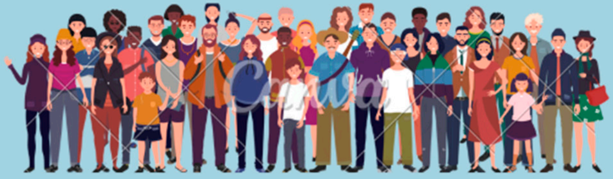

Scopriamo in che modo...

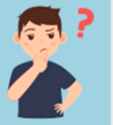

Sapere di quali vaccini hai bisogno: assicurati che tu e la tua famiglia siate vaccinati in tempo, ogni volta

Imparare insieme: conoscete i fatti e condividete con i vostri amici o conoscenti la verità sui benefici della vaccinazione, allontanando la disinformazione

Coinvolgere la tua rete: chiedi ai tuoi amici di sostenere la Settimana mondiale dell'immunizzazione per promuovere i vaccini

WORLD HEALTH ORGANIZATION 2025

RIFLETTIAMO INSIEME...

Perché la vaccinazione non è solo una questione di salute personale, ma anche di salute pubblica?

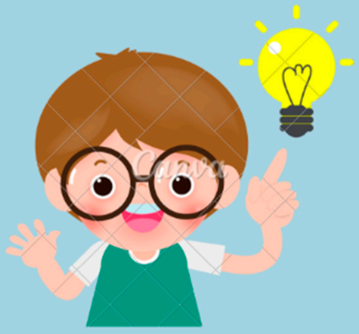

Qual è lo strumento utilizzato per eliminare una malattia infettiva?

Perchè è importante vaccinarsi?

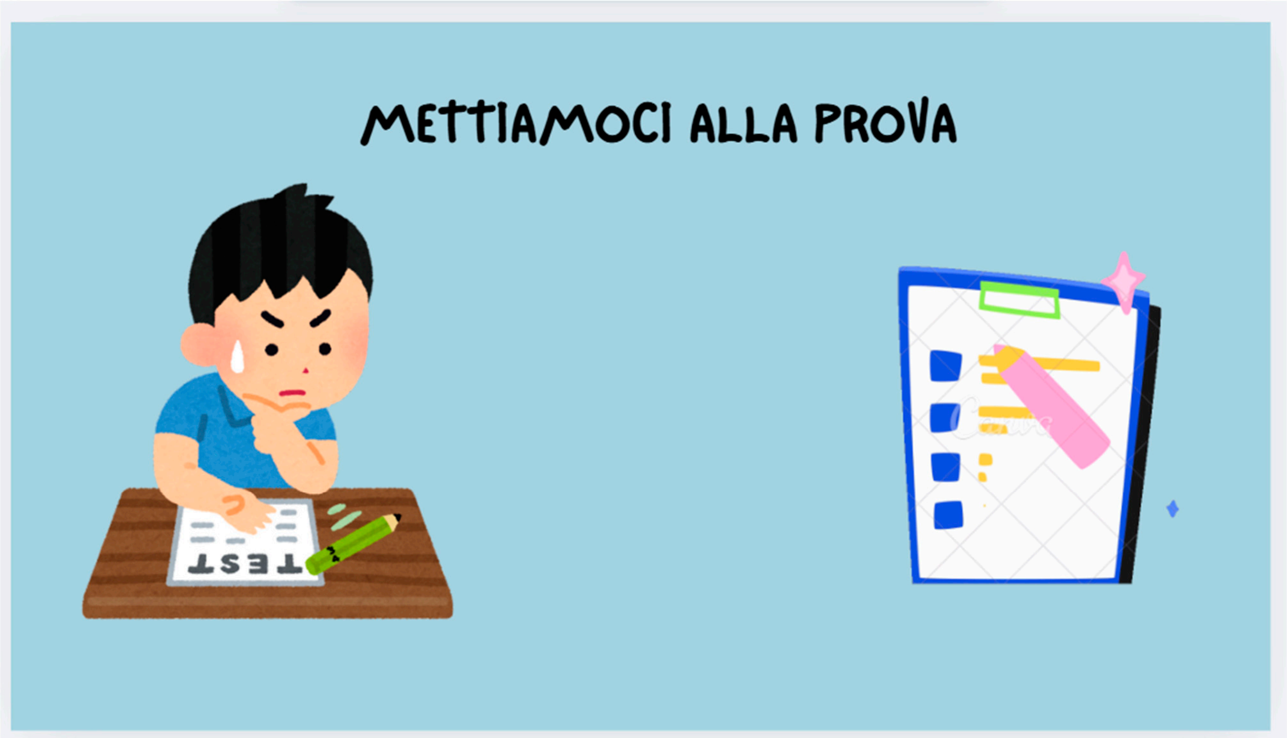
METTIAMOCI ALLA PROVA
TEST

Summary slide

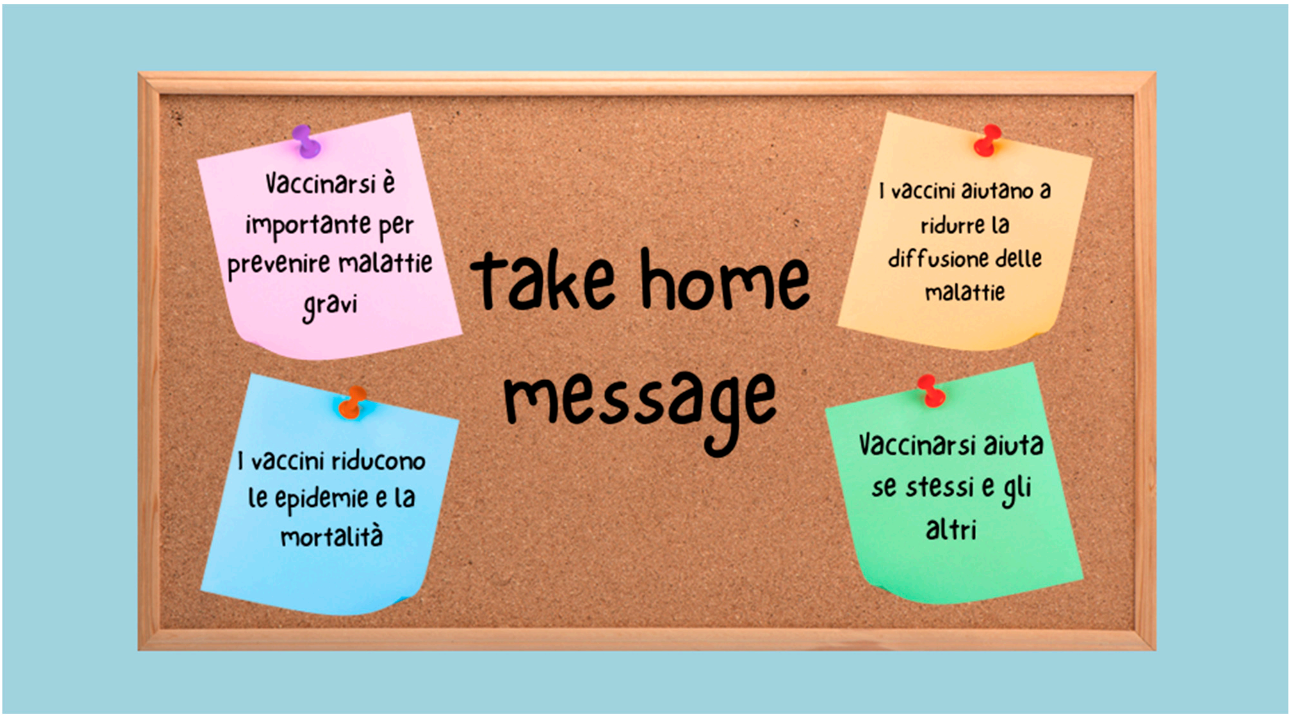

Online brochure

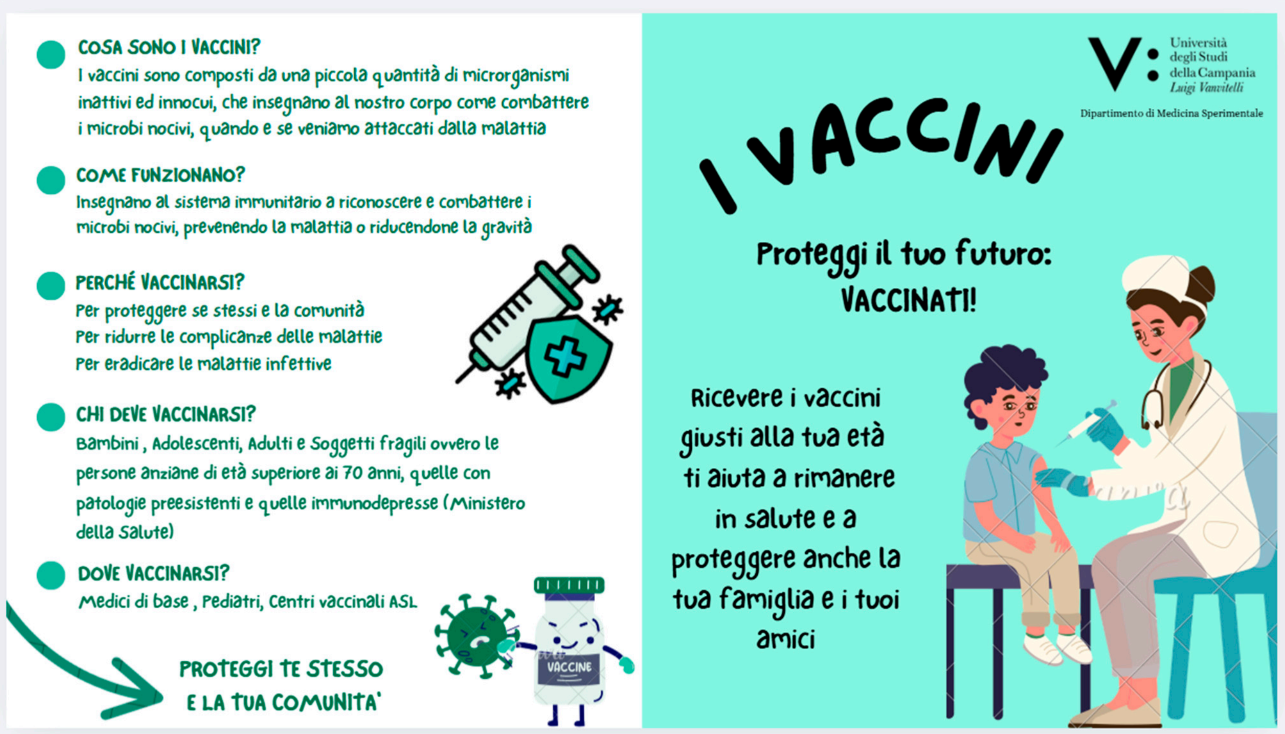

Supplement: Supplementary file 1 [file vaccines-14-00368-s001.zip › File S2.pdf]
