# Supplementary figures and images for: Improving Vaccine Knowledge Among Adolescents Aged 11–14 Years: A Pre–Post School-Based Educational Intervention
Source: Vaccines (Basel). 2026 Apr 22;14(5):368. doi: 10.3390/vaccines14050368 (PMC13211575; doi:10.3390/vaccines14050368)

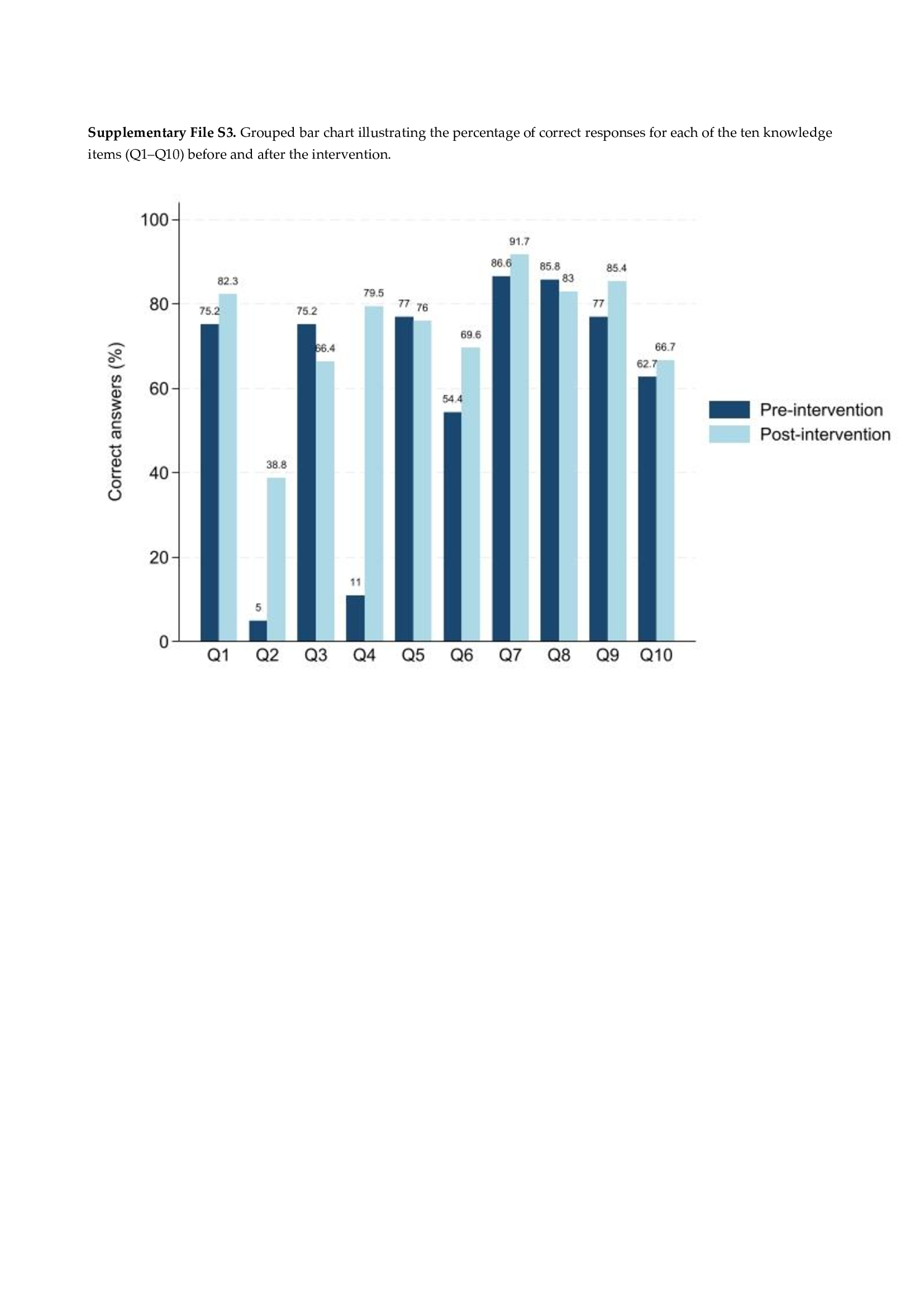

Supplement: Supplementary file 1 [file vaccines-14-00368-s001.zip › File S3.jpg]

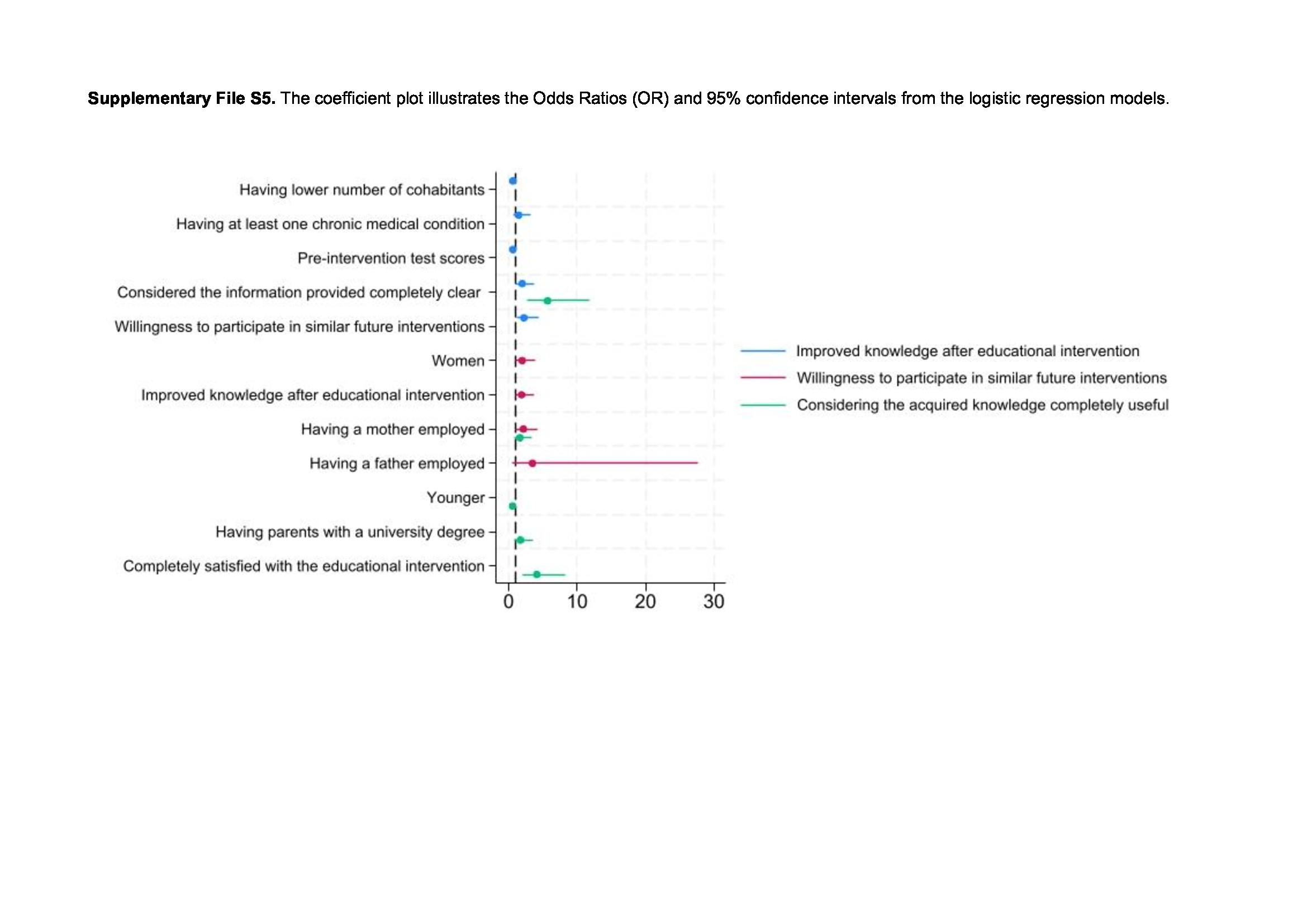

Supplement: Supplementary file 1 [file vaccines-14-00368-s001.zip › File S5.jpeg]

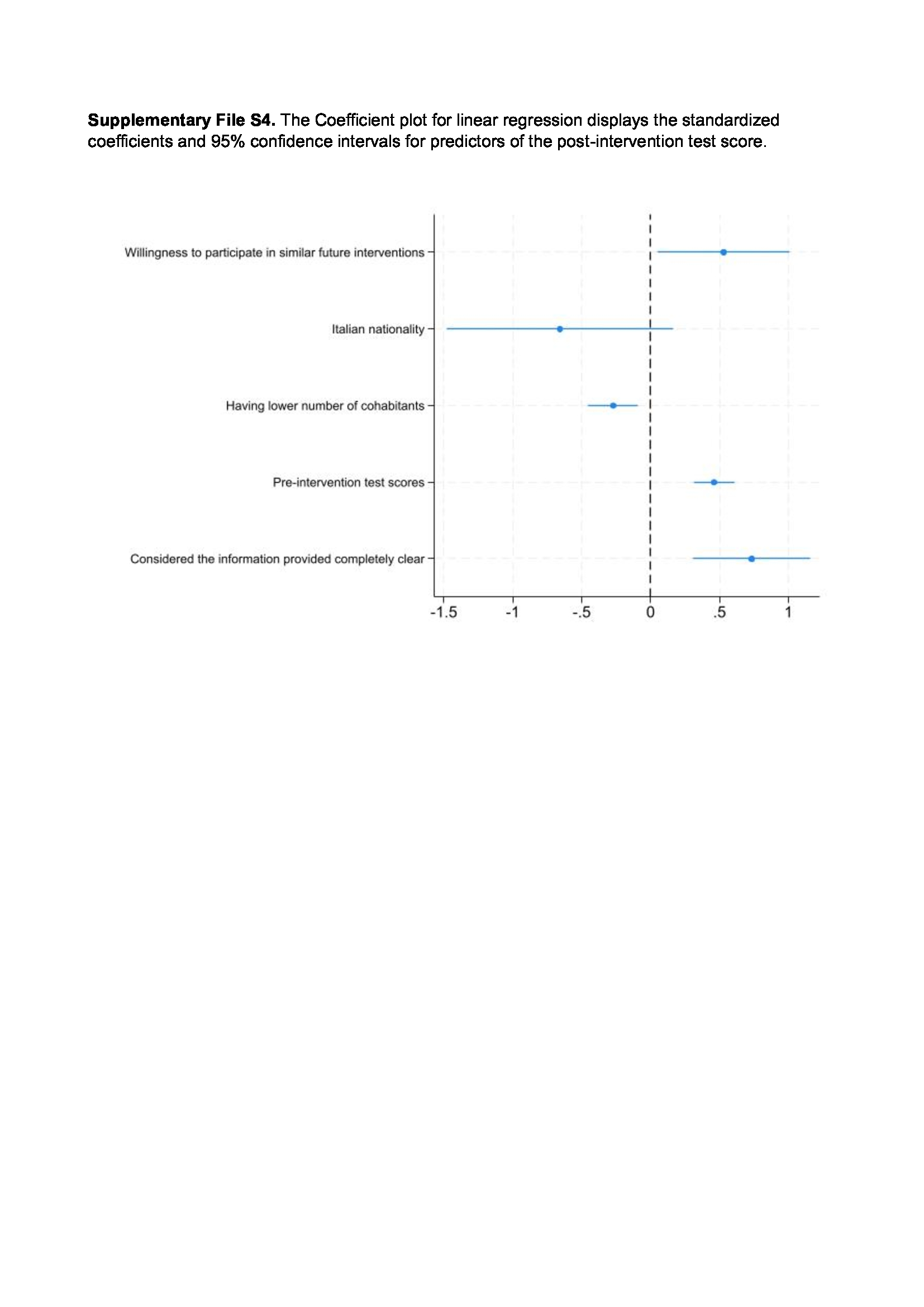

Supplement: Supplementary file 1 [file vaccines-14-00368-s001.zip › File-S4.jpeg]
